# Suppressive effect of secretory phospholipase A_2 _inhibitory peptide on interleukin-1β-induced matrix metalloproteinase production in rheumatoid synovial fibroblasts, and its antiarthritic activity in hTNFtg mice

**DOI:** 10.1186/ar2810

**Published:** 2009-09-18

**Authors:** Maung-Maung Thwin, Eleni Douni, Pachiappan Arjunan, George Kollias, Prem V Kumar, Ponnampalam Gopalakrishnakone

**Affiliations:** 1 Department of Anatomy, Yong Loo Lin School of Medicine, 4 Medical Drive, National University of Singapore, 117597 Singapore; 2 Institute of Immunology, Biomedical Sciences Research Center, Alexander Fleming, 34 Al. Fleming Street, 16672 Vari, Greece; 3 Porter Neuroscience Research Center, NEI/NIH, 35 Lincoln Drive, MSC 3731, Bethesda, Maryland 20892, USA; 4 Department of Orthopaedic Surgery, Yong Loo Lin School of Medicine, 4 Medical Drive, National University of Singapore, 117597 Singapore

## Abstract

**Introduction:**

Secretory phospholipase A_2 _(sPLA_2_) and matrix metalloproteinase (MMP) inhibitors are potent modulators of inflammation with therapeutic potential, but have limited efficacy in rheumatoid arthritis (RA). The objective of this study was to understand the inhibitory mechanism of phospholipase inhibitor from python (PIP)-18 peptide in cultured synovial fibroblasts (SF), and to evaluate its therapeutic potential in a human tumor necrosis factor (hTNF)-driven transgenic mouse (Tg197) model of arthritis.

**Methods:**

Gene and protein expression of sPLA_2_-IIA, MMP-1, MMP-2, MMP-3, MMP-9, tissue inhibitor of metalloproteinase (TIMP)-1, and TIMP-2 were analyzed by real time PCR and ELISA respectively, in interleukin (IL)-1β stimulated rheumatoid arthritis (RA) and osteoarthritis (OA) synovial fibroblasts cells treated with or without inhibitors of sPLA2 (PIP-18, LY315920) or MMPs (MMP Inhibitor II). Phosphorylation status of mitogen-activated protein kinase (MAPK) proteins was examined by cell-based ELISA. The effect of PIP-18 was compared with that of celecoxib, methotrexate, infliximab and antiflamin-2 in Tg197 mice after ip administration (thrice weekly for 5 weeks) at two doses (10, 30 mg/kg), and histologic analysis of ankle joints. Serum sPLA_2 _and cytokines (tumor necrosis factor (TNF)α, IL-6) were measured by *Escherichia coli *(*E coli*) assay and ELISA, respectively.

**Results:**

PIP-18 inhibited sPLA_2_-IIA production and enzymatic activity, and suppressed production of MMPs in IL-1β-induced RA and OA SF cells. Treatment with PIP-18 blocked IL-1β-induced p38 MAPK phosphorylation and resulted in attenuation of sPLA_2_-IIA and MMP mRNA transcription in RA SF cells. The disease modifying effect of PIP-18 was evidenced by significant abrogation of synovitis, cartilage degradation and bone erosion in hTNF Tg197 mice.

**Conclusions:**

Our results demonstrate the benefit that can be gained from using sPLA_2 _inhibitory peptide for RA treatment, and validate PIP-18 as a potential therapeutic in a clinically relevant animal model of human arthritis.

## Introduction

Rheumatoid arthritis (RA) is a chronic inflammatory condition that is considered to be one of the more common and difficult to treat autoimmune diseases. Although the biologic agents (e.g., monoclonal antibodies to TNF and IL-6 receptor, and recombinant soluble TNFα receptor, etc.) can achieve significant suppression of the complex inflammatory network and ameliorate the disease, they are still subject to the general disadvantages associated with protein drugs, such as insufficient immune response to infectious agents and autoimmunity [1,2]. Therefore, further development of molecular agents that target the specific intracellular pathways that are activated in RA synovium would offer an attractive therapeutic option.

Besides cytokines, chemokines, adhesion molecules and matrix degrading enzymes that are responsible for synovial proliferation and joint destruction [3], phospholipase A_2 _(PLA_2_), a key enzyme in the production of diverse mediators of inflammatory conditions, is also implicated in the pathophysiology of RA [4]. Among the vast family of PLA_2 _enzymes, which includes three cellular (cPLA_2_) isoforms and 10 secretory PLA_2 _(sPLA_2_) isoforms (IB, IIA, IIC, IID, IIE, IIF, III, V, X, and XII), group IIA secretory phospholipase (sPLA_2_-IIA) is proinflammatory *in vivo *[5]. It is an attractive target in RA because it releases arachidonic acid from cell membranes under some conditions, enhances cytokine induction of prostaglandin (PGE) production, and is associated with enhanced release of IL-6 [6]. Proinflammatory cytokines and sPLA_2 _potentiate each other's synthesis, thereby creating an amplification loop for propagation of inflammatory responses [7]. Hence, inhibition of sPLA_2 _may logically block the formation of a wide variety of secondary inflammatory mediators.

In our search for such an inhibitor, we designed a 17-residue peptide (P-NT.II) using the parent structure of the protein termed Phospholipase Inhibitor from Python serum (PIP) [8,9]. We have already shown proof of the concept that this small molecule sPLA_2 _inhibitory peptide P-NT.II has a disease-modifying effect particularly evident on cartilage and bone erosion with eventual protection against joint destruction [10]. In our recent study, we designed several analogs of P-NT.II and their inhibitory activity was evaluated by *in vitro *inhibition assays against a purified human synovial sPLA_2 _enzyme. Using cell-based assays, gene and protein expression analyses, along with nuclear magnetic resonance and molecular modeling-based investigations, we have demonstrated that a linear 18-residue peptide PIP-18 potently inhibits IL-1β-induced secretions of sPLA_2 _and matrix metalloproteinases (MMPs; 1, 2, 3, and 9) in RA synovial fibroblasts (SF), at protein and mRNA levels [11].

As sPLA_2 _[2,4] and MMPs [12] have been proposed to play a significant role in RA etiology, such peptide inhibitors may be effective and beneficial for the treatment of RA. However, despite their potential utility in human diseases, both inhibitors have limited efficacy in RA to date [13-15]. Improvements in therapeutic benefit may be achieved by targeting both sPLA_2 _and MMPs. Here, we extended our study to examine the therapeutic efficacy of PIP-18 on a clinically relevant TNF-driven transgenic mouse model of human RA [16], and to study the possible mechanism of peptide inhibition of the inflammatory pathway in human RA SF.

## Materials and methods

### Clinical specimens

Synovial tissues were collected from the knee joints of RA (n = 5) or osteoarthritis (OA; n = 5) patients at total knee-replacement surgery and used for primary cultures within one hour after collection. Informed consent was taken from the patients with RA or OA who were diagnosed according to the 1987 revised clinical criteria of the American College of Rheumatology [17]. All samples were collected at the National University Hospital, Department of Orthopaedic Surgery, National University of Singapore, according to the guidelines of the Institutional Review Board.

### Synovial fibroblast cell cultures

SF cells were isolated from the tissues by enzymatic digestion with 1 mg/ml of collagenase II (Worthington Biochemical Corporation, Lakewood, NJ, USA) for 20 minutes at 37°C, and cultured under standard conditions (37°C/5% carbon dioxide (CO_2_)) in DMEM supplemented with 10% FBS, 100 U/ml of penicillin, and 100 mg/ml of streptomycin (Gibco-BRL products, Gaithersburg, MD, USA). Cells were passaged by trypsin digestion and split at a ratio of 1:3. Confirmation of more than 90% purity of SF cell populations at passages three and onwards involved staining for prolyl 4 hydroxylase (5B5 antibody, Abcam, Cambridge, MA, USA) and fluorescence-activated cell sorting analysis. Cells were washed and plated in DMEM, and only passages three to five were used in our cell-based studies. For experiments, confluent SF cells were serum-starved overnight and the medium was then replaced with fresh serum-free DMEM containing 0.5% sterile-filtered, cell culture grade BSA (Sigma-Aldrich, St. Louis, MO, USA) as a carrier protein. Three different doses (1, 5, or 10 μM) of PIP-18 were examined to find the peptide concentration that showed maximal inhibitory effect on IL-1β-induced sPLA_2 _production. SF cells were preincubated for one hour with 5 μM of PIP-18, a selective sPLA_2 _inhibitor LY315920 (Lilly Research Laboratories, Indianapolis, IN, USA), MMP Inhibitor II (Merck Singapore Pte Ltd., Singapore), or with vehicle (0.5% dimethyl sulfoxide (DMSO)), and then stimulated with 10 ng/ml of human recombinant (hr)IL-1β (Chemicon, Temecula, CA, USA) for 24 hours. SFs cultured without IL-1β or the peptide served as controls.

### Cell viability assays

XTT (Sodium 3'- [Phenyl amine carboxyl)-3, 4-tetrazolium]-bis (4-methoxy-nitro) benzene sulfonic acid hydrate) Cell Proliferation Kit II (Roche Applied Science, Indianapolis, IN, USA) was used to assess the possible cytotoxic effect of the peptides on the human RA/OA SF cells.

### Immunoassays and cell-based ELISA

RA/OA SF samples were centrifuged briefly, and supernatants were stored at -20°C until used. To assess the concentration of secreted proteins, supernatants of RA/OA SF primary cultures were analyzed in triplicate, using commercially available kits for sPLA_2 _(sPLA_2 _human type IIA enzyme-linked immunoassay kit, Cayman Chemical Co., Ann Arbor, MI, USA), MMP-1, MMP-2, MMP-3, MMP-9, tissue inhibitor of matrix metalloproteinase (TIMP)-1 and -2 (RayBiotech, Inc., Norcross, GA, USA). Analysis of serum levels of human TNFα and murine IL-6 was undertaken using ELISA (R&D Systems, Minneapolis, MN, USA). Phosphorylation of mitogen-activated protein kinase (MAPK) proteins was examined using SuperArray CASE™ cell-based ELISA kit [18], and specific MAPK inhibitors (p38 inhibitor SB202190, Erk inhibitor PD98059, and Jun N-terminal Kinase (JNK) inhibitor SP600125 (all from SuperArray Bioscience Corporation, Frederick, MD, USA) as positive controls.

### Escherichia coli-based sPLA_2 _assay

Mouse serum sPLA_2 _levels were measured as described [10] with minor modifications. Briefly, reaction mixtures (250 μl) containing 25 mM CaCl_2_-100 mM Tris/HCl (pH 7.5) assay buffer, [^3^H] arachidonate-labeled *Escherichia coli *membrane (5.8 μCi/μmol, PerkinElmer Life Sciences, Inc, MA, USA) suspension in assay buffer (about 10,000 counts per minute (cpm)) and 10 μl of the serum diluted (1:50) in assay buffer containing 0.1% fatty-acid-free BSA (Sigma-Aldrich, St. Louis, MO, USA) were incubated for one hour at 37°C. The reaction was terminated with 750 μl of chilled PBS containing 0.1% fatty-acid-free BSA. The undigested substrate was pelleted by centrifugation at 12,000 g for five minutes, and aliquots (500 μl) of the supernatant taken for measurement of the amount of [^3^H] arachidonate released from the *E. coli *membrane using liquid scintillation counting (LS 6500 Scintillation Counter; Beckman Inc., CA, USA). Standard assay conditions were set up prior to sPLA_2 _determination in mouse serum. The linear range for sPLA_2_-containing mouse serum was first established by serial dilution of pooled mouse serum, while that of the standard curve was determined with the purified secreted sPLA_2_-IIA human recombinant protein (GenWay Biotech, Inc., CA, USA). To find out any possible influence of the serum components on sPLA_2 _standard curve, a fixed volume of 1:50 diluted mouse serum was added into varying amounts (1 to 200 ng/ml) of purified sPLA_2 _standard before the assay. Diluting the mouse serum samples by at least 50-fold with the assay buffer containing 0.1% fatty-acid-free BSA attained a linearity range of 1 to 80 ng/ml of sPLA_2_. The amount of sPLA_2 _present in the serum was calculated from the standard curve (ng/ml sPLA_2 _on X-axis *versus *cpm/ml on Y-axis) and is expressed as ng/ml ± standard error of the mean.

### Quantitative real-time RT-PCR

After removal of supernatants for protein assays, the remaining SF cells were washed with cold PBS, and pooled (n = 3 flasks) for each group: - IL-1β, + IL-1β, IL-1β + PIP-18, IL-1β + LY315920, and IL-1β + MMP II. Total RNA was isolated using RNeasy^® ^mini kit (Qiagen, Inc., Valencia, CA, USA), subsequently treated with RNase-free Dnase-I (Qiagen Inc., Valencia, CA, USA) at 25°C for 20 minutes, and stored at -80°C until used. The quality (A_260_/A_280 _ratio = 1.9 to 2.1) and quantity of extracted RNA were determined by spectrophotometry (Bio-Rad Laboratories, Hercules, CA, USA). Reverse transcription of RNA, amplification, detection of DNA, data acquisition, primer design, and quantitative real-time PCR analysis were all performed as described [19]. PCR primers (forward/reverse) for sPLA_2_-IIA, MMP-1, MMP-2, MMP-3, MMP-9, TIMP-1, TIMP-2 and glyceraldehyde 3-phosphate dehydrogenase (GAPDH; 1^st ^BASE Pvt. Ltd., Singapore) were as follows: (5'-AAGGAAGCCGCACTCAGTTA-3')/(5'-GGCAGCAGCCTTATCACACT-3'); (5'-AC-AGCTTCCCAGCGACTCTA-3')/(5'-CAGGGTTTCAGCATCTGGTT-3'); (5'-TTGACGGTAAGGACGGACTC-3')/(5'-ACTTGCAGTACTCCCCATCG-3'); (5'-GAGGACACCAGCATGAACCT-3')/(5'-CACCTCCAGAG-TGTCGGAGT-3'); 5'-CTCGAACTTTGACAGCGACA-3'/5'-CCCTCAGTGAAGCGGTACAT-3'; 5'-TGACA-TCCGGT TCGTCTACA-3'/5'-CACTGTGCATTCCTCACAGC-3'; 5'-GATGCACATCACCCTCTGTG-3'/5'-GTGCCCGTTGATGTTCTTCT-3'; 5'-CAAGGTCATCCACGACCACT-3'/5'-CCAGTGAGTTTCCCGTTCAG-3'. GAPDH expression was used as an internal calibrator for equal RNA loading and to normalize relative expression data for all other genes analyzed. The real-time PCR data were quantified using relative quantification (2^-ΔΔC^T) method [20].

### Experimental animals

Heterozygous human TNF-transgenic mice (strain Tg197; in a mixed genetic background C57BL/6xCBA), bred and maintained in the animal facility at the Biomedical Sciences Research Centre, Fleming, Greece, were used to evaluate the effectiveness of the peptide PIP-18 as compared with other drugs. In these mice, a chronic inflammatory and destructive polyarthritis develops within three to four weeks after birth [21]. All mouse procedures were conducted in compliance with the institutional guidelines.

### Drugs used in animal studies

Methotrexate (Sigma-Aldrich, St. Louis, MO, USA), infliximab (Remicade, Schering-Plough Labo N.V., Belgium), celecoxib (Pfizer Inc, New York, NY, USA), and antiflammin-2 (custom synthetised peptide) were used as comparators to the lead anti-inflammatory peptide P-NT.II and optimized analog PIP-18. All peptides were custom synthesized by AnaSpec, Inc, San Jose, CA, USA, at a purity of more than 95%.

### Drug treatment

Ten weight-matched groups of Tg197 mice (n = 8 per group; statistically calculated with a power (1 - β) of 90% and a significance level (α) of 5%) were injected intraperitoneally (three times a week for five weeks) with various drugs at age three weeks (arthritis onset). Two different doses (10 and 30 mg/kg) were used to examine the effect of peptides (P-NT.II and PIP-18) on experimental arthritis. Except for methotrexate, which was used at a lower dose of 1 mg/kg due to its higher toxicity, doses of 10 mg/kg were used for infliximab, celecoxib, and antiflammin-2 peptide (AF-2). These doses were selected according to those prespecified in the available literature and according to our studies of other rodents in *in vivo *models [21-24].

### Clinical and histopathologic assessments

Body weight and arthritic scores (AS) were recorded weekly for each mouse. Evaluation of arthritis in ankle joints was peformed in a blinded manner using a semiquantitative AS ranging from 0 to 3 as described previously [10]. At eight weeks of age all mice were killed by CO_2 _inhalation, and the hind ankle joints removed for histology. Histologic processing, scoring and analytical assessments of ankle joints are carried out basically, as previously described [10,21].

### Statistical analysis

Unless otherwise indicated, the analysis of variance (ANOVA) single-factor test was used to evaluate group means of continuous variables. If the ANOVA single-factor test was significant, a *post hoc *test was performed using a Bonferroni's correction. Analyses were performed using Prism statistical software (GraphPad Prism version 4.01, GraphPad Software Inc., San Diego, CA, USA).

## Results

### Composition of RA and OA synovial fibroblasts

Table 1 shows that an average of 75% of the RA and OA SF cells at the first passage were fibroblasts (Prolyl-4-hydroxylase +; mAb 5B5, Dianova, Hamburg, Germany) and 15% were macrophages (CD14+; mAb Tyk4, Dako, Hamburg, Germany), while T cells (CD-3+; mAb UCHT-1, ATCC, Manassas, VA, USA) and B cells (CD 20+; mAb B-Ly1, Dako, Hamburg, Germany) represent less than 1% of the SF cells. Starting from the third passage and onwards, on average approximately 99% of the SF cells were fibroblasts, with very few (< 1%) contaminating macrophages, T cells and B-cells detected by fluorescence-activated cell sorting analysis.

**Table 1:** Percentage of fibroblasts and contaminating cells in primary cultures of RA and OA synovial fibroblast cells at various passages

Passage	Cell type	% positive cells (Mean ± SEM)*	
		
		RA SF	OA SF
First	Fibroblast (Prolyl-4-hydroxylase +)^1^	75 ± 8.0	68 ± 5.0
	Monocyte/macrophage (CD14+)^2^	15 ± 2.0	21 ± 3.5
	T-cells (CD3+)^3^	0.8 ± 0.2	1.2 ± 0.3
	B-cells (CD20+)^4^	0.9 ± 0.3	0.8 ± 0.2
			
Third	Fibroblast (Prolyl-4-hydroxylase +)	99 ± 0.5	98.5 ± 0.6
	Monocyte/macrophage (CD14+)	0.8 ± 0.2	0.6 ± 0.1
	T-cells (CD3+)	0.5 ± 0.1	0.8 ± 0.2
	B-cells (CD20+)	0.6 ± 0.2	0.5 ± 0.1
			
Fourth	Fibroblast (Prolyl-4-hydroxylase +)	98 ± 0.4	99.2 ± 0.4
	Monocyte/macrophage (CD14+)	1.0 ± 0.5	0.95 ± 0.3
	T-cells (CD3+)	0.5 ± 0.2	0.5 ± 0.1
	B-cells (CD20+)	0.9 ± 0.1	0.8 ± 0.1

* Total number = five rheumatoid arthritis (RA) and five osteoarthritis (OA) patients. Monoclonal antibodies used for flow cytometry: mAb 5B5 ^1^; mAb Tyk4 ^2^; mAb UCHT-1 ^3^; mAb B-Ly1 ^4^. SEM = standard error of the mean; SF = synovial fluid.

### Suppression of secreted sPLA2 and MMPs

**Figure 1:**
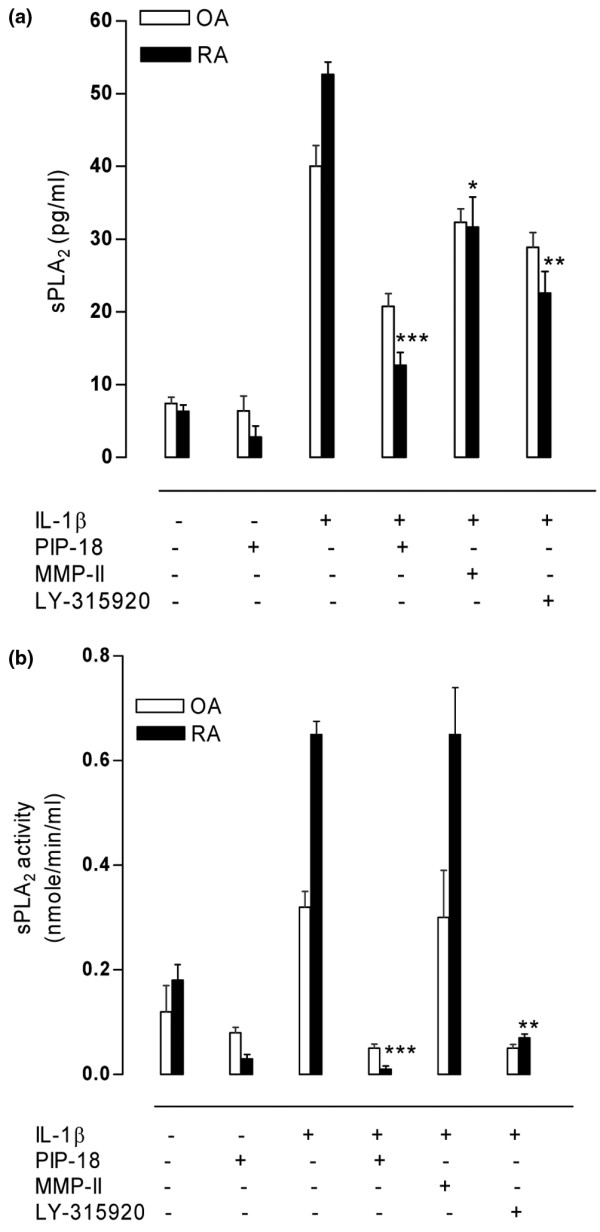
Inhibition of sPLA_2_-IIA release into medium by PIP-18 in RA and OA SF cultures. Confluent synovial fibroblast (SF) cells in 75 cm^2 ^flasks were serum-starved for overnight (16 hours) before incubation for one hour with 5 μM PIP-18, LY315920, matrix metalloproteinase inhibitor II (MMP-II), or with vehicle (0.5% dimethyl sulfoxide final concentration in medium), and stimulation with hrIL-1β (10 ng/ml) for 24 hours. Rheumatoid arthritis (RA)/osteoarthritis (OA) SFs cultured without IL-1β or the inhibitors served as controls. **(a) **Immunoreactive secretory phospholipase A_2 _(sPLA_2_) released in the culture medium was determined by sPLA_2 _human type IIA enzyme-linked immunoassay kit. **(b) **sPLA_2 _enzymatic activity was measured with an *Escherichia coli *membrane assay as described [11]. Data shown are the mean ± standard error of the mean of the combined data of triplicate determination of triplicate experiments performed on a pool of RA SF cultures from five RA patients. One-way analysis of variance with *post hoc *test was done using Bonferroni's correction. *P < 0.05, **P < 0.001, ***P < 0.001 for pair-wise comparisons of each inhibitor type (IL without inhibitor *versus *IL with inhibitor). PIP = phospholipase inhibitor from python.

**Figure 2:**
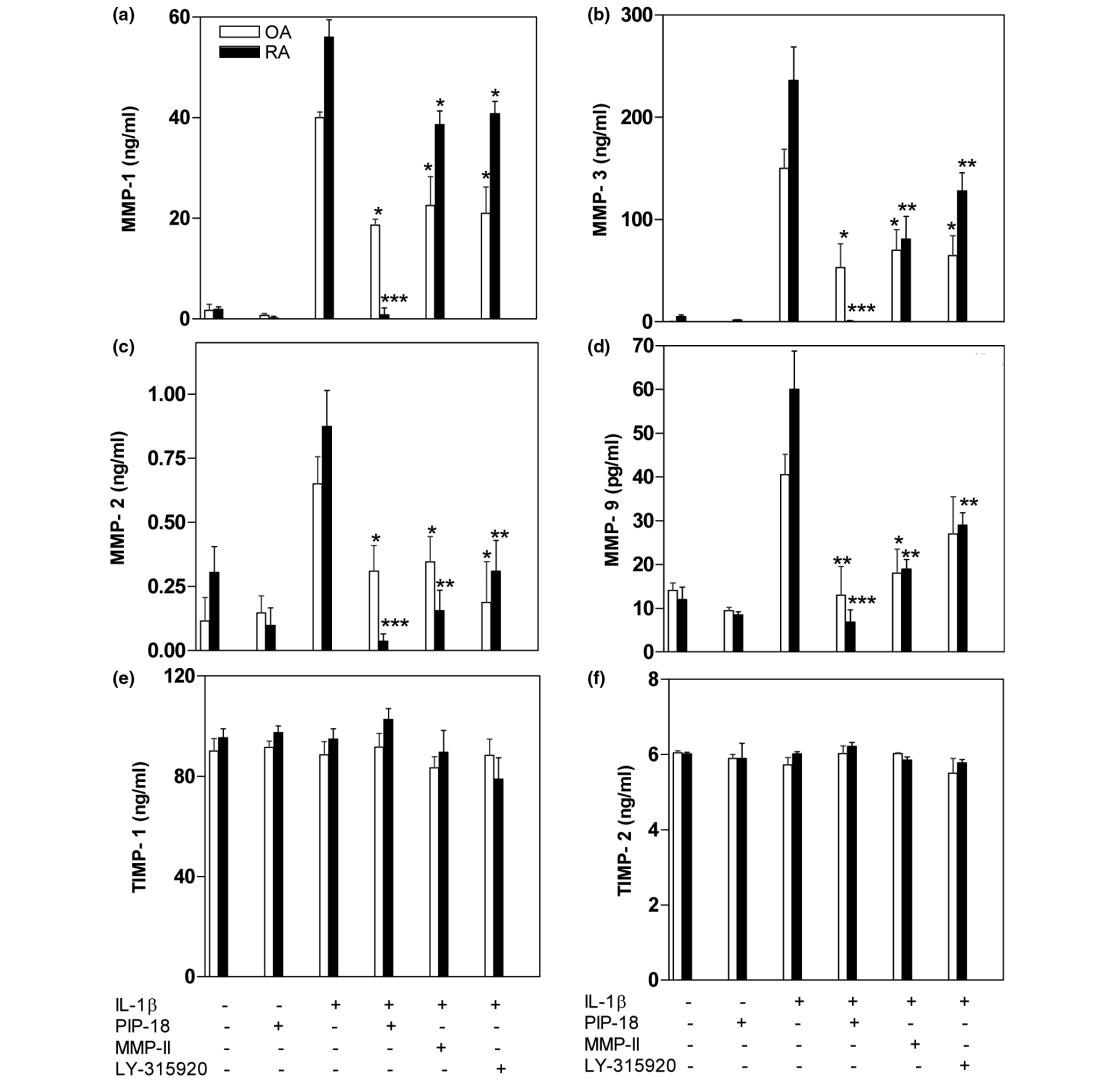
Suppressive effects of PIP-18 *versus *sPLA_2 _and MMP inhibitors on MMP secretion. Osteoarthritis (OA) and rheumatoid arthritis (RA) synovial fibroblast (SF) cells were incubated for one hour with 5 μM phospholipase inhibitor from python (PIP)-18, matrix metalloproteinase (MMP)-II inhibitor or secretory phospholipase A_2_(sPLA_2_) inhibitor LY-315920, stimulated overnight with rhIL-1β (10 ng/ml), and supernatants assayed for MMP secretions by ELISA: **(a) **MMP-1, **(b) **MMP-3, **(c) **MMP-2, **(d) **MMP-9, **(e) **tissue inhibitor of metalloproteinase (TIMP)-1, **(f) **TIMP-2. Results are the mean ± standard error of the mean of the combined data of triplicate determination of triplicate experiments done on a pool of RA SF cultures from five RA patients. Bonferroni's post hoc test was done only if the analysis of variance single-factor test was found significant. *P < 0.05, **P < 0.01, ***P < 0.001 for pair-wise comparisons (IL without inhibitor *versus *IL with each of the inhibitor used in the study.

The suppressive effect of PIP-18, LY315920 [25] and MMP inhibitor II [26] on IL-1β-stimulated sPLA_2 _and MMP protein expression was examined in human RA and OA SF cultures. The peptide used at 1 to 10 μM was nontoxic to the cells after 24 hours treatment, and hence 5 μM (IC_50 _of PIP-18) was applied in our cell-based assays to study its effect. The release of sPLA_2_-IIA in the medium by unstimulated cells was barely detectable, but was markedly increased by nearly 10-fold and 8-fold by IL-stimulated RA and OA SF cells, respectively. Elevated sPLA_2 _production was significantly suppressed more by PIP-18 (***P < 0.001) than LY315920 (**P < 0.01), while MMP inhibitor II was the least (*P < 0.05) effective (Figure 1a). As compared with unstimulated controls, significantly augmented sPLA_2 _activity (*P *< 0.001) was detected in the culture media of IL-stimulated cells recovered after 24 hours incubation. Pretreatment of those cells with PIP-18 or LY 315920 significantly (***P < 0.001, *vs *IL alone) reduced this elevated activity, whereas no significant inhibition of sPLA_2 _activity (*P *> 0.05) was noted in the cells pretreated with MMP-II (Figure 1b). Consistent with the increased sPLA_2 _secretion by IL-1β-stimulated SF cells, marked production of MMPs (MMP-1, MMP-2, MMP-3 and MMP-9) was also observed at 24 hours (Figure 2). This IL-induced MMP production was significantly suppressed by one hour of pretreatment of SFs with PIP-18 (***P < 0.001), or to a lesser degree with LY315920 (**P < 0.01). None of the inhibitors had any effect on TIMP-1 and TIMP-2 productions.

### Suppression of sPLA2 and MMP transcription

**Figure 3:**
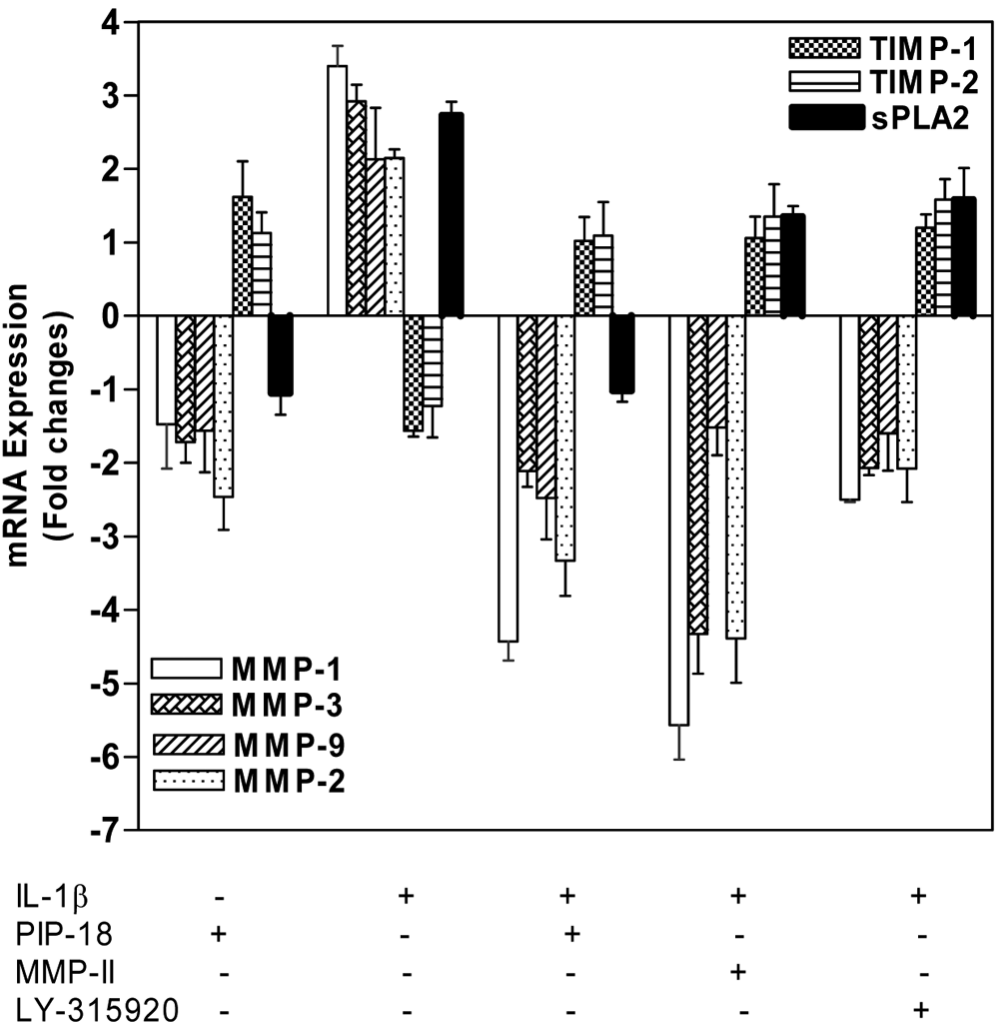
Peptide treatment inhibited MMP and sPLA_2 _gene expression in IL-1β induced RA SF. Cells were pretreated with the peptide (phospholipase inhibitor from python (PIP)-18), secretory phospholipase A_2 _(sPLA_2_) inhibitor (LY315920) or matrix metalloproteinase inhibitor (MMP-II) at 5 μM for one hour, and incubated with hrIL-1β (10 ng/ml) for 24 hours before isolating total RNA. Relative mRNA expression levels were determined by real-time PCR analyses, normalized to internal *GAPD *values, and plotted relative to control samples treated with vehicle (0.5% dimethyl sulfoxide). Gene-specific real-time analysis was performed for all seven mRNA targets, sPLA2, MMP-1, -2, -3, -9, tissue inhibitor of metalloproteinase (TIMP)-1 and TIMP-2. Results shown are the mean ± standard deviation of fold inductions from three independent experiments with a pool of rheumatoid arthritis (RA) synovial fibroblast (SF) cultures obtained from five RA patients.

Quantitative RT-PCR was used to assess relative mRNA expression levels of IL-1β-induced human RA SF in the presence and absence of PIP-18 (Figure 3). More than a 1.5-fold increase or decrease of each gene relative to GAPDH was taken as a significant change [27]. Transcription of MMP-1 (3.4 fold), MMP-2 (2.1 fold), MMP-3 (2.9 fold), MMP-9 (2.13 fold), and sPLA_2 _(2.73 fold) was significantly upregulated except for TIMP-1 (-1.4 fold) and TIMP-2 (-1.23 fold), which were downregulated to levels that were not statistically significant (< -1.5 fold) following stimulation with IL-1. Comparison of the results between the PIP-18-treated and untreated SFs indicates that significant inhibition of gene expression was evident in human RA SF for MMP-1, -2, -3, -9, and sPLA_2_, but not for TIMP-1 and TIMP-2. In contrast, sPLA_2_-IIA expression in LY315920-treated RA SF did not differ significantly from that of untreated cells, indicating that it is not as robust as PIP-18 effect on sPLA_2 _expression.

### PIP-18-mediated inhibitory effect is signaled through p38 MAPK

**Figure 4:**
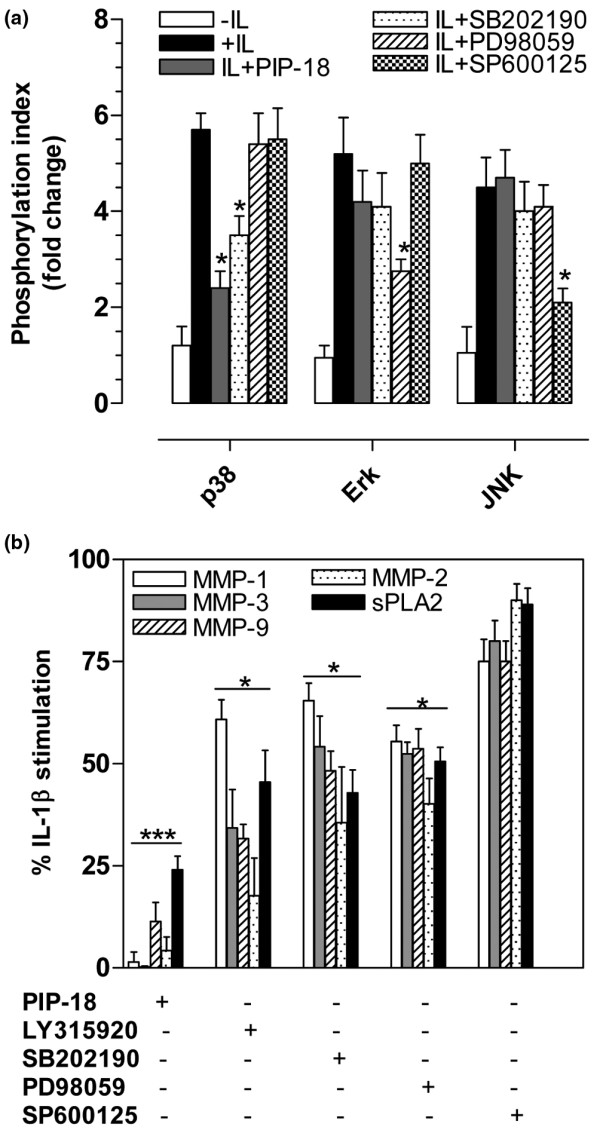
PIP-18 suppresses IL-stimulated p38 MAPK phosphorylation. **(a) **Rheumatoid arthritis (RA) synovial fibroblast (SF) cells were preincubated at 37°C for one hour with various inhibitors at optimal concentrations: phospholipase inhibitor from python (PIP)-18 (5 μM), LY315920 (5 μM), SB202190 (10 μM), PD98059 (1 μM) or SP600125 (5 μM), and stimulated with rhIL-1β (10 ng/ml) for 30 minutes before assaying for p38, Erk and JNK phosphorylation, using cell-based ELISA. For control of systematic variation, blank control wells (without cells) as well as experimental control wells (seeded cells without any treatment) were included. Phosphorylation index (Pi) was calculated as relative levels of the phosphorylated form of mitogen-activated protein kinase (MAPK)/total MAPK levels. Values are mean ± standard error of the mean (SEM) of three separate experiments presented as fold increase of Pi of experimentally treated cells relative to control cells without any treatment. **(b) **RA SF from separate experiments were pretreated with inhibitors as in (a), followed by stimulation with hrIL-1β (10 ng/ml) for 16 hours, and supernatants analyzed for secretory phospholipase A_2_(sPLA_2_) and matrix metalloproteinase (MMPs) as indicated. Values expressed as % IL-1β stimulation are mean ± SEM for four experiments for each condition. PIP-18 was more effective in suppressing MMP/sPLA_2 _production (***P < 0.001 *vs *IL), while LY315920, p38 and Erk inhibitors were relatively less effective (*P < 0.05 *vs *IL). *P < 0.05, **P < 0.01 (one-way analysis of variance with Bonferroni's *post hoc *test); for pair-wise comparisons (IL without inhibitor *versus *IL with each of the inhibitor used in the study).

The phosphorylation status of MAPK proteins in IL-1β-stimulated RA SF cells before and after treatment with the peptide or specific MAPK inhibitors is shown in Figure 4a. Phosphorylation of MAPK proteins (p38, Erk, and JNK) was significantly increased to 5.7 ± 0.55, 5.2 ± 0.75, and 4.9 ± 0.62 folds (mean ± standard error), respectively upon stimulation with IL-1β (*P *< 0.05, *vs *unstimulated). Pretreatment of RA SF cells with either of the specific inhibitors SB202190, PD98059, or SP600125, significantly (*P < 0.05 *vs *IL) inhibited phosphorylation of p38, Erk, and JNK, respectively. p38 phosphorylation was specifically inhibited only by its specific inhibitor SB202190 (*P *< 0.05, *vs *IL), but not by Erk inhibitor PD98059 or JNK inhibitor SP600125. PIP-18 selectively and significantly reduced IL-1β-induced p38 phosphorylation from 5.7 ± 0.55 to 2.4 ± 0.35-fold (*P < 0.05, *vs *IL). Erk phosphorylation was only partially reduced from 5.2 ± 0.75 to 4.2 ± 0.65-fold (*P *> 0.05, *vs *IL), while the peptide had little or no effect on JNK phosphorylation (*P *> 0.05, *vs *IL). These findings collectively indicate that PIP-18 exerts its effect on the MAPK signaling pathway via attenuation of p38 phosphorylation.

The effects of sPLA_2 _inhibitors (PIP-18 and LY315920) and MAPK inhibitors (SB202190, PD98059, SP600125) on IL-1β-induced MMP and sPLA_2 _production by RA SF are shown in Figure 4b. sPLA_2 _inhibitors as well as inhibitors of p38 and Erk, significantly suppressed MMP and sPLA_2 _secretion. PIP-18 was more effective in suppressing MMP/sPLA_2 _production to less than 20% of the control levels (***P < 0.001 *vs *IL), while LY315920, p38 and Erk inhibitors were relatively less effective (*P < 0.05 *vs *IL). With the JNK inhibitor SP600125, no significant (*P *> 0.05) effect was found on MMP or sPLA_2 _production.

### Impact of PIP-18 on arthritis progression

**Figure 5:**
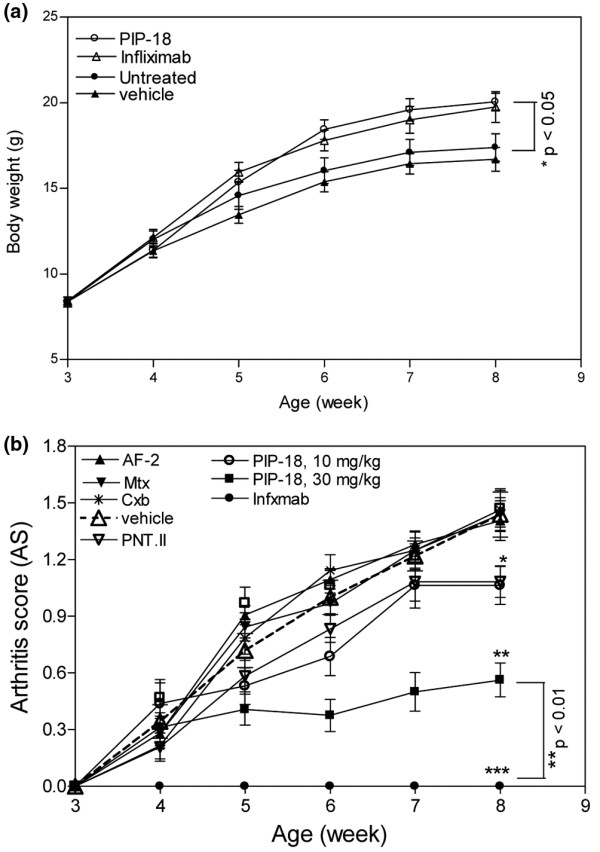
Beneficial effects of PIP-18 on disease outcome. Intraperitoneal injections commenced at age three weeks and terminated at eight weeks. Body weights were recorded before and weekly after injections. **(a) **Tg197 mice injected with phospholipase inhibitor from python (PIP)-18 (30 mg/kg) or infliximab (10 mg/kg) significantly (*P < 0.05, *vs *untreated) gained body weights at eight week. Drugs without effect are not shown. **(b) **Low dose (10 mg/kg) of peptides shows effect at eight weeks, while the higher dose of PIP-18 (30 mg/kg) or infliximab (10 mg/kg) effectively reduced arthritis score (AS) at six weeks. AS was significantly reduced at eight weeks in the ankle joints of mice treated with 10 mg/kg of P-NT.II or PIP-18 (*P < 0.05 vs untreated), and 30 mg/kg of PIP-18 (**P < 0.01, vs untreated) or 10 mg/kg of infliximab (***P < 0.001, vs untreated). Data are mean ± standard error of the mean of 16 joints per group (One-way analysis of variance with Bonferroni's multiple comparison test).

The clinical effect was assessed based on the body weight gain and the degree of swelling and deformation of the ankle joints of Tg197 mice. As compared with untreated or vehicle-treated mice, only the groups that received 30 mg/kg of PIP-18 and 10 mg/kg of infliximab had significant increase (*P *< 0.05 relative to untreated animals) in body weights at eight weeks of age, while the remaining groups of mice did not show any significant weight gain during the five-week study course (Figure 5a).

AS obtained during the five-week-treatment period (Figure 5b) showed a marked suppression of disease progression in mice treated with the peptides (10 mg/kg P-NT.II or 10 to 30 mg/kg of PIP-18) or 10 mg/kg infliximab, but not in untreated Tg197 mice or those treated with vehicle (DMSO), AF-2, methotrexate, or celecoxib. AS taken at terminal point (Figure 5b) indicated that PIP-18 (30 mg/kg) or infliximab (10 mg/kg) had the maximal suppressive effect on disease progression (**P < 0.001, *vs *untreated or vehicle treated). Treatment with lower doses of peptide (10 mg/kg of P-NT.II or PIP-18) also significantly (*P < 0.01, *vs *untreated) reduced AS, but had less impact on disease progression as compared with treatment with a higher PIP-18 dose (30 mg/kg). Infliximab (10 mg/kg) was significantly more effective than 30 mg/kg PIP-18 (**P < 0.01) in reducing AS (two-tailed paired *t*-test).

### Histopathologic evidence of peptide-mediated disease modulation

**Figure 6:**
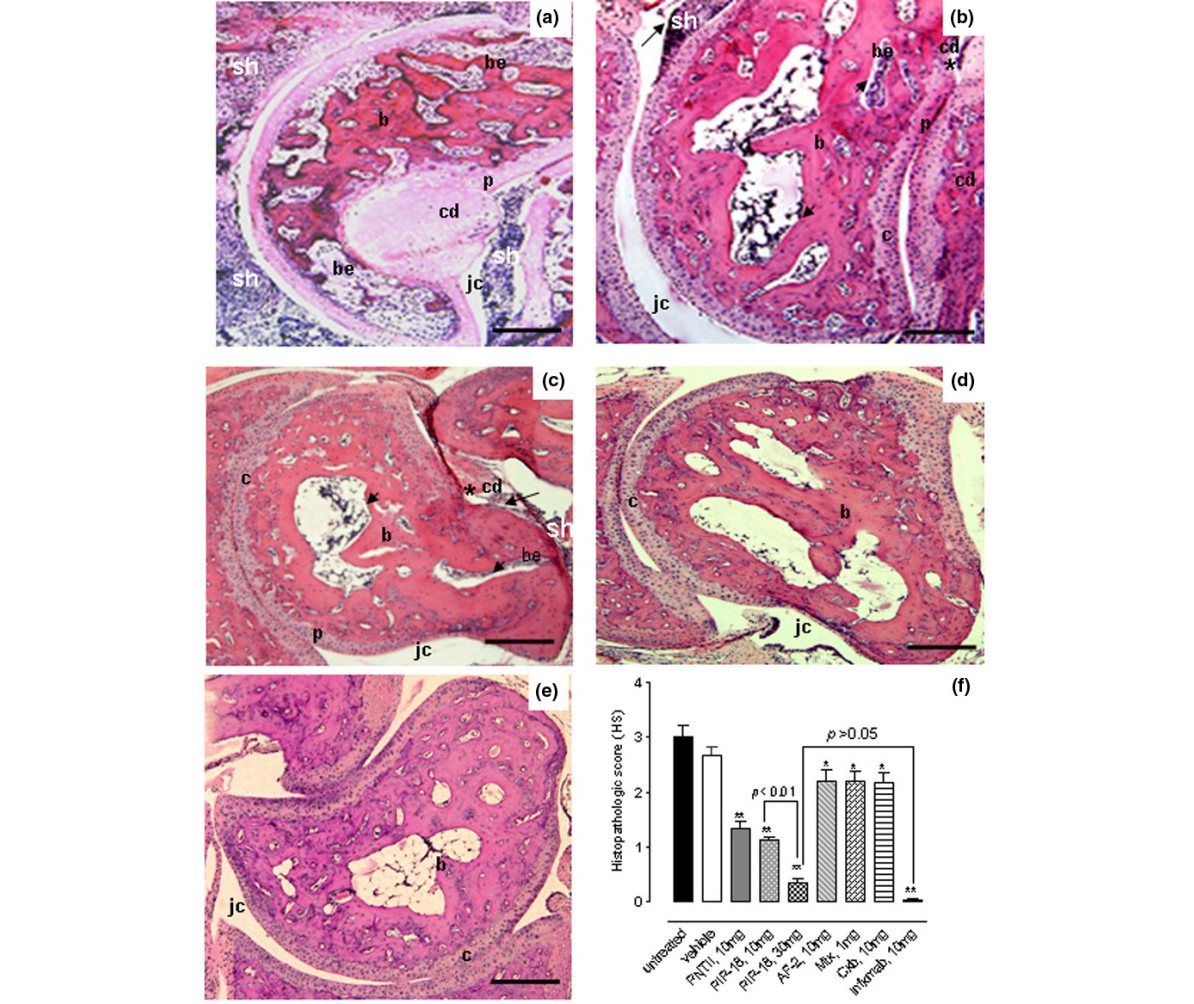
Histopathologic evidence of peptide-mediated disease modulation. H&E-stained representative ankle sections from Tg197 mice **(a) **without treatment, or after treatment with **(b) **10 mg/kg and **(c) **30 mg/kg of phospholipase inhibitor from python (PIP)-18, respectively for five weeks (n = 16 joints/group). The extent of synovial hyperplasia (sh), cartilage degradation (cd), and bone erosion (be) was less marked in the joints of (b, c) peptide-treated group than in (a) untreated joints, with histologic appearance more or less similar to that seen in the **(d) **infliximab treated or **(e) **normal (wild type) joints. Note the less marked hyperplasia (arrow), cartilage destruction (*) and bone erosion (arrowhead) in the representative joint of (c) 30 mg/kg PIP-18-treated group compared with that of (b) 10 mg/kg PIP-18-treated group. b = bone; be = bone erosion; c = cartilage; cd = cartilage degradation; jc = joint cavity; sh = synovial hyperplasia. **(f) **Mean histopathologic scores (HS) are shown for different treatment groups. Compared with untreated mice, P-NT.II, PIP-18 and infliximab treatment significantly decreased HS (**P < 0.001) as did treatment with antiflammin-2, methotrexate (Mtx), and celecoxib (Cxb), which were less effective (*P < 0.01). Higher dose (30 mg/kg) of PIP-18 was more effective than the lower dose (10 mg/kg) (*P < 0.01). One-way analysis of variance with Bonferroni's multiple comparison post test. Bars = 500 μm. Infliximab (10 mg/kg) and 30 mg/kg PIP-18 had similar modulatory effect on HS (*P *> 0.05, two-tailed paired *t*-test).

Synovitis and joint histopathology as shown in the representative tissue sections from Tg197 ankle joints (Figure 6) indicate that the joints of the untreated, vehicle-treated or those treated with methotrexate, celecoxib, or AF-2 were moderately to severely damaged by the expansion of synovial pannus and destruction of cartilage and bone structures (Figure 6a). The beneficial effect of peptide treatment on synovial inflammation, cartilage and bone erosions was evident at 10 mg/kg (Figure 6b), with the effect becoming more pronounced at a higher dose of 30 mg/kg (Figure 6c). No marked difference was seen in the histologic features between the joints of mice treated with 30 mg/kg PIP-18 (Figure 6c) and 10 mg/kg infliximab (Figure 6d), with joint pathology appears to be similar to that of normal (wildtype) joint (Figure 6e) in both cases. As shown in the graph (Figure 6f), histopathologic score values obtained for the two groups (30 mg/kg PIP-18 *vs *10 mg/kg infliximab) were not significantly different (*P *> 0.05, two-tailed paired *t*-test). There was a significant reduction in the mean histopathologic score in joints of mice that received 30 mg/kg of PIP-18 or 10 mg/kg of infliximab (**P < 0.01), 10 mg/kg of P-NT.II or PIP-18 (**P < 0.01), 1 mg/kg of methotrexate, and 10 mg/kg celecoxib or AF-2 (*P < 0.05) when compared with the joints of the untreated control Tg197 (Figure 6f).

### PIP-18 modulates joint inflammation and bone destruction more favorably than DMARDs

**Figure 7:**
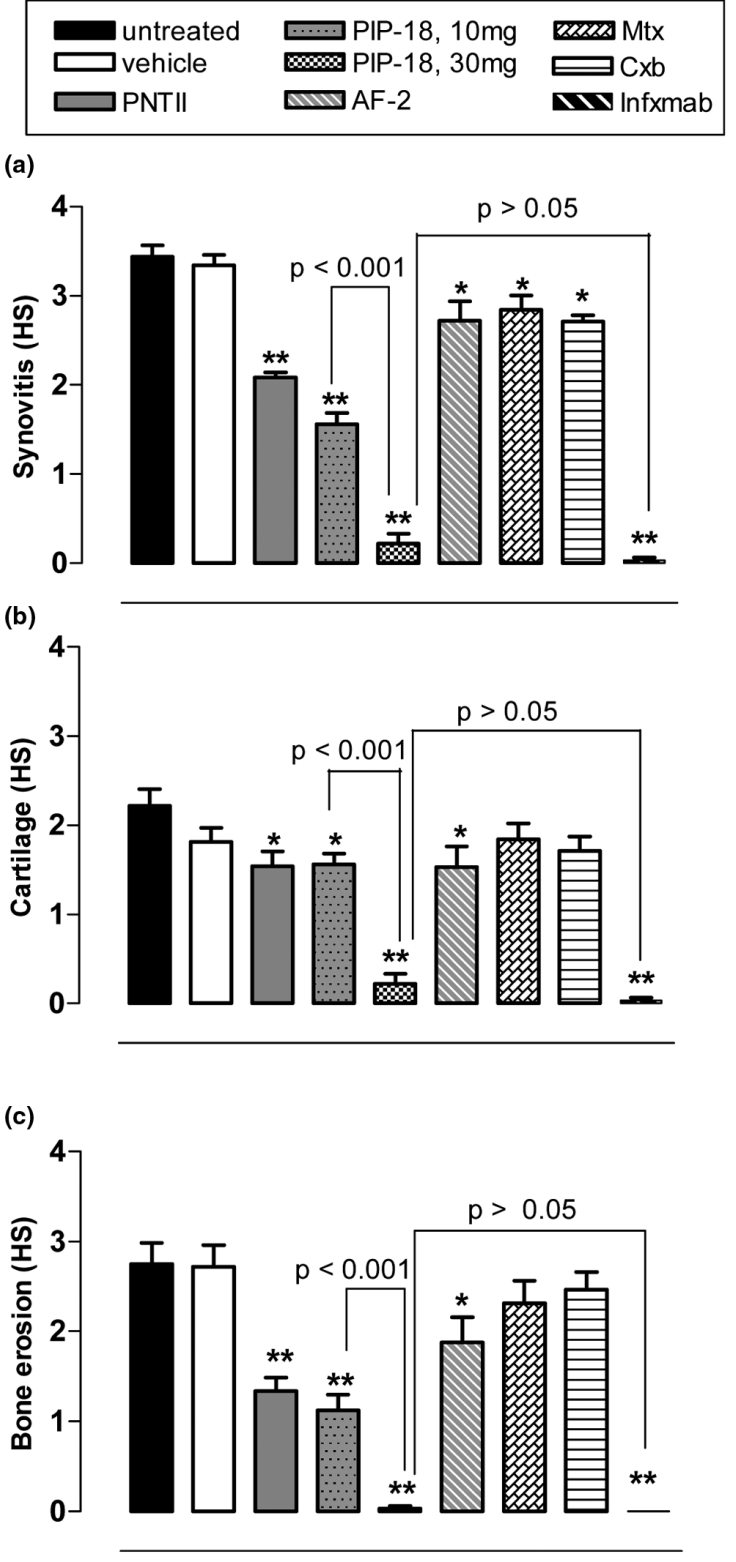
PIP-18 modulates joint inflammation and bone destruction more favorably than AF-2 peptide and DMARDs. Differential histologic scores (HS) of ankle joints of untreated Tg197 mice or those treated with the peptides (P-NT.II and phospholipase inhibitor from python (PIP)-18) or comparator drugs (methotrexate (Mtx); celecoxib (Cxb); infliximab (infxmab); antiflammin-2 (AF-2)) are shown. Compared with other drugs, infliximab and the peptides P-NT.II and PIP-18 significantly inhibited **(a) **synovitis, **(b) **cartilage destruction and **(c) **bone erosion. DMARD = disease-modifying anti-rheumatic drug.

Administration of PIP-18 at doses of 30 mg/kg three times per week for five weeks in Tg197 mice resulted in a significant reduction (**P < 0.01) in all three analytical histopathologic scores (synovitis, cartilage destruction and bone erosion) as compared with those of untreated Tg197 mice, which all developed synovitis with severe articular cartilage degradation and bone erosions (Figures 7a to 7c). Comparative analyses showed PIP-18 to be more potent than the disease-modifying anti-rheumatic drugs (DMARDs; methotrexate and celecoxib) or the anti-inflammatory peptide (AF-2) in suppressing synovitis, cartilage degradation and bone erosion. Methotrexate and celecoxib are the DMARDs that are presently used for arthritis treatment. As compared with PIP-18, both drugs are less effective in reducing synovitis (Figure 7a) or cartilage (Figure 7b) and bone (Figure 7c) components of arthritis in our transgenic mouse model. PIP-18 peptide was more potent than the DMARDs (methotrexate and celecoxib) or the anti-inflammatory peptide (one way ANOVA with Bonferroni's multiple comparison post test; *P < 0.01, **P < 0.001 *vs *untreated control), and was as effective as infliximab in suppressing synovitis, cartilage degradation and bone erosion (*P *> 0.05, two-tailed paired *t*-test).

### Serum levels of sPLA2 and proinflammatory cytokines

**Figure 8:**
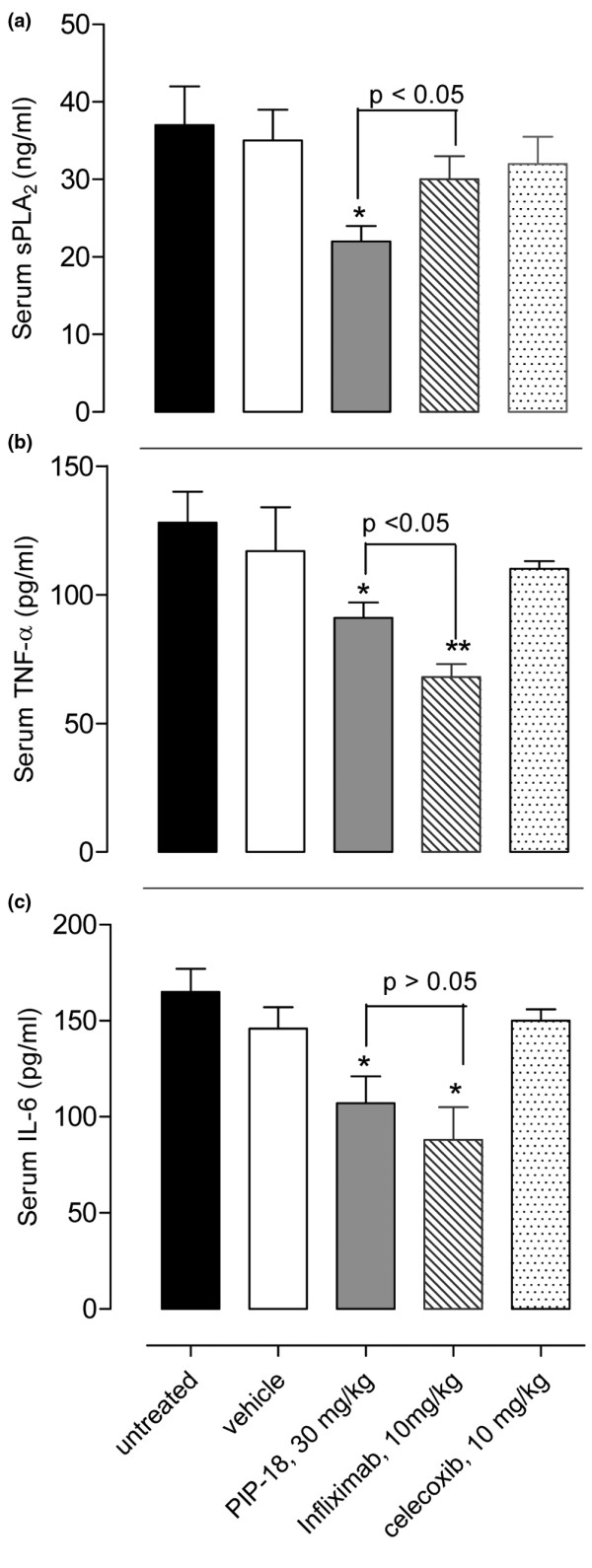
Serum levels of murine sPLA_2 _and IL-6, and human TNF-α. Tg197 mice received either vehicle (0.5% dimethyl sulfoxide in phosphate-buffered saline), peptides (P-NT.II or PIP-18), or comparator drugs (antiflammin-2, methotrexate, celecoxib and infliximab) at age three weeks (disease onset), and blood samples collected by cardiac puncture at termination (age eight weeks). Murine (m) serum secretory phospholipase A_2 _(sPLA_2_) levels were measured with an *Escherichia coli *membrane assay. Analysis of murine TNF-α and IL-6 was done by ELISA. Values are the mean ± standard error of the mean of each group; *P < 0.05; **P < 0.01 *vs *untreated or vehicle treated Tg197 mice.

Compared with untreated or vehicle-treated Tg197 mice, serum levels of murine sPLA_2 _and IL-6, (msPLA_2_, mIL-6), and human TNF (hTNF-α) decreased significantly (*P < 0.05 *vs *untreated) at five-week post-treatment with 30 mg/kg PIP-18 (Figure 8). Infliximab (10 mg/kg) significantly reduced serum hTNF-α ((**P < 0.01) and mIL-6 ((*P < 0.05) levels, but had no significant (*P *> 0.05) effect on msPLA_2_. In contrast, none of the serum levels of msPLA_2_, mIL-6 and hTNF-α were significantly reduced in mice treated with celecoxib. Other peptides (P-NT.II or AF-2) or methotrexate that did not show any significant changes, were excluded from Figure 8 for clarity.

## Discussion

Despite the initial success seen with the use of small molecule inhibitors of sPLA_2 _and MMPs in animal models [28,29], interests in their therapeutic potential have been mitigated by undesirable side effects [30] and a lack of efficacy [13,14,31] observed in later clinical trials. Compared with MMP inhibitors, sPLA_2 _inhibitors have a better safety profile, but have limited efficacy in clinical studies [14,15]. One of the potential reasons for the failure of LY333013 may be incomplete inactivation of sPLA_2 _in the SF due to inadequate dose of the inhibitor used in the trial [32]. As sPLA_2 _and MMP inhibitors have limited efficacy in RA, the use of an inhibitor that can target both sPLA_2 _and MMP could be advantageous.

In our study, inhibition of sPLA_2 _production and mRNA expression is reflected by a significant decrease of sPLA_2 _enzymatic activity in IL-induced RA SF cells pretreated with PIP-18. In contrast to LY315920, a small molecule that binds directly to the sPLA_2 _active site for inhibition [33], a 2000 Dalton PIP-18 peptide is proposed to bind to the hydrophobic binding pocket near the N-terminal helix of sPLA_2 _[11]. PIP-18 has two putative pharmacophores for binding more than one molecule of sPLA_2_, and this may account for its relatively stronger suppressive effect on sPLA_2 _transcription and translation as compared with that of LY315920. The strong inhibitory effect of PIP-18 on enzymatic activity as well as protein and mRNA expression of sPLA_2 _may perhaps be a unique feature of this peptide. It inhibited more than 70% of sPLA_2 _secretion and more than 90% of mRNA expression in IL-induced RA SF cells, suggesting that the inhibitory effect of PIP-18 on sPLA_2 _occurs at transcriptional and post-transcriptional levels. To provide a comprehensive picture of the inhibitory effect of different inhibitors on cytokine-stimulated expression of sPLA_2 _and MMP genes and secreted proteins in RA and OA SF cells, we acknowledge here that part of the data previously published elsewhere [11] have been incorporated in Figures 1 to 3 of this paper.

In normal human synoviocytes, sPLA_2_-IIA steady-state mRNA is inducible by IL-1 [4], whereas in human RA SF, IL-1-β does not appear to induce sPLA_2_-IIA protein and enzyme activity [34]. The data on sPLA_2_-IIA steady-state mRNA reported herein are conclusive because they are obtained with very sensitive quantitative RT-PCR techniques, thus confirming our finding that sPLA_2_-IIA mRNA is indeed inducible by IL-1 in cultured human RA and OA SF cells. Although our data appears to be at odds with the previous report [34], the relevance of our data on IL-induced sPLA_2_-IIA protein secretion in RA SF cells may be supported by the fact that sPLA_2_-IIA protein is detectable by immunofluorescence in synovial fibroblast cells from RA patients [35].

As sPLA_2 _has previously been suggested as a regulator of MMP activation [36], the effect of PIP-18 on MMPs seems only secondary to sPLA_2 _inhibition. The suppressive effect of PIP-18 on sPLA_2 _and MMP transcription found in IL-induced RA SF (Figure 3) may likely be due to its interference on transcription factors like MAPKs, one of the several potential targets for therapeutic intervention in RA [37]. As nuclear factor (NF)-*κ*B is also implicated in MMP transcription [12], its involvement in PIP-18-mediated MMPs suppression, although not reported herein, could not be ruled out. Compared with JNK and extracellular signal-regulated kinase (ERK), p38 MAPK is strongly activated by IL-1β stimulation, and is highly susceptible to PIP-18 inhibition, suggesting that the effect of peptide on MMP transcription is related to its ability to modulate the activation of the p38 MAPK pathway in RA SF cells. Although JNK and ERK specific inhibitors are known to block IL-1-β-induced MMP expression in cultured cells, we did not find any significant inhibition of MMPs with SP 600125 or PD 98059 in our cell-based studies (Figure 4b). The failure to block cytokine-induced expression of MMPs by SP 600125 or PD 98059 inhibitors has also been reported in other studies [38-40]. Because small molecule MMP inhibitors targeting MMP enzymatic activity are known to cause side effects in clinical trials [30], modulating MMP gene expression as an alternative to targeting MMP enzymes will offer a better strategy of controlling inflammatory joint diseases such as RA.

Of note, some differences between PIP-18 and LY315920 are evident with respect to their ability to suppress different MMPs in IL-1β-induced RA SF (Figure 4b). The MMP inhibition potency of PIP-18 is in the order, MMP3>MMP1~MMP2~MMP9, whereas that of LY315920 is MMP2>MMP9~MMP3>MMP1 (Figure 4b), suggesting that the two sPLA_2 _inhibitors may not be identical in their mode of action. Differential regulation of MMP-3, MMP-2, and MMP-9 has been reported with respect to the ERK, JNK, and p38 MAPK pathways [41]. IL-1β-stimulated production of MMP-3 and -1 in RA SFs is suppressed by specific p38 MAPK inhibitors [42,43]. MMP-2 expression is relatively less sensitive to MAPK inhibition than MMP-3 and MMP-1, due to the absence of binding sites for activator protein 1 (AP-1) transcription factor in the MMP-2 promoter [44]. Hence, it is likely that PIP-18 appears to mediate IL-1β-induced expression and synthesis, particularly of MMP-3 and MMP-1, at the level of transcription involving p38 MAPK and AP-1, while LY315920 may exert its effect via mediation of different transcriptional pathways or other regulatory mechanisms.

**Figure 9:**
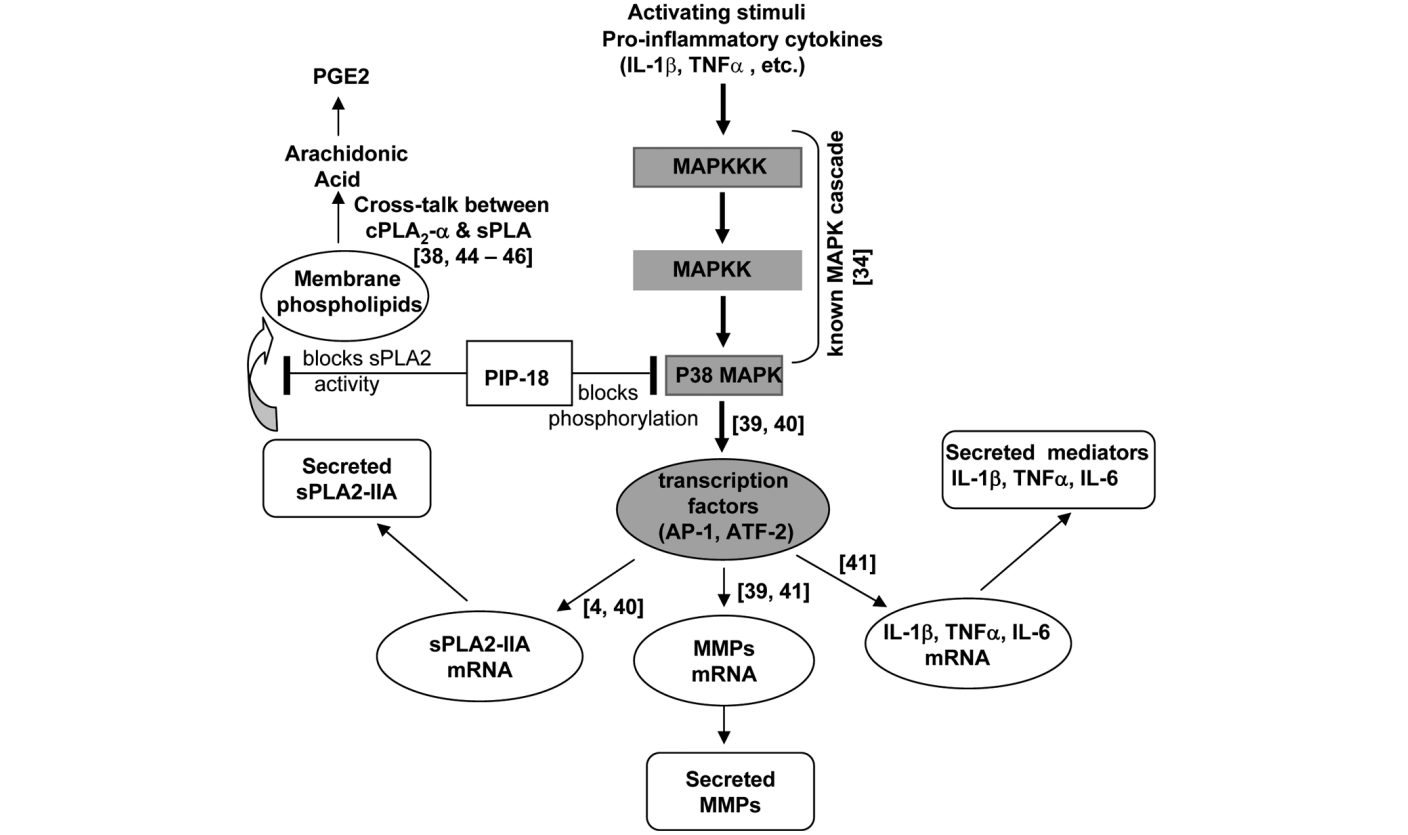
Possible mechanism of PIP-18 suppression on IL-stimulated expression of sPLA_2 _and MMPs. IL-1β and/or TNF initiate the expression of secretory phospholipase A_2 _(sPLA_2_)-IIA and matrix metalloproteinases (MMP) through activation of mitogen-activated protein kinase (MAPK) cascade. (1) phospholipase inhibitor from python (PIP)-18 blocks p38 MAPK phosphorylation and reduces activation of transcription factors (activator protein-1 (AP-1), activating transcription factor 2 (ATF-2)), which regulate the transcription of sPLA_2_-IIA, MMPs (MMP-1, MMP-2, MMP-3, MMP-9) and proinflammatory cytokines (IL-6, TNF, IL-1). This results in downregulation of these genes and decreased protein secretions. (2) Inhibition of sPLA_2 _enzymatic activity by PIP-18 contributes to reduced generation of arachidonic acid for prostaglandin production. MAPKKK = MAPK kinase kinase; MAPKK = MAPK kinase; PGE2 = prostaglandin E2; sPLA_2_-IIA = secretory phospholipase A_2_-Group IIA; **solid arrows**, known pathways; ┤, inhibition (NF-κB pathway is not shown here).

The possible mechanism by which PIP-18 peptide suppresses cytokine-stimulated expression of sPLA_2 _and MMP genes and secreted proteins is depicted in Figure 9. In this proposed model, PIP-18 binds sPLA_2 _and inhibits its enzymatic activity, leading to reduced PGE_2_production. sPLA_2_-IIA enzymatic activity is required to amplify cytokine-stimulated PGE_2 _production in cultured RA SF [4,35], and it has been reported that sPLA_2 _inhibitors, LY311727 [4] and a cyclic peptide [45], effectively block sPLA_2_-IIA-mediated amplification of cytokine-induced PGE_2 _production in cultured RA SF through inhibition of sPLA_2_-IIA enzymatic activity. Besides inhibiting sPLA_2 _activity, PIP-18 also blocks p38 MAPK phosphorylation. These results suggest that sPLA_2 _inhibition and blocking of p38 MAPK activation by PIP-18 are independent functions, and may support the view that PIP-18 is a dual-function inhibitor.

Based on well-known pathways (as indicated by solid lines in Figure 9), IL-1β and/or TNF initiate the expression of sPLA_2_-IIA and MMPs through activation of MAPK cascade involving MAPKKK, MAPKK and MAPKs [37]. p38 MAPK contributes to transcription of MMPs and sPLA_2_-IIA by promoting expression of AP-1 genes [46,47]. According to our results, PIP-18 blocks mainly IL-induced p38 MAPK phosphorylation, which may result in the diminished available pool of activated AP-1, possibly leading to reduced mRNA expression and decreased secretion of sPLA_2_, MMPs, and cytokines [46-48]. The proinflammatory cytokines have the ability to stimulate all four p38 MAPK isoforms [49], but there are differences among the isoforms with respect to the mode of activation, substrate specificity, and function [50]. As the present data do not provide information on the differential effect of PIP-18 on p38 isoforms, it would be interesting to direct our future research on that aspect.

Besides, it is also possible that blocking p38 MAPK activity by PIP-18 may diminish cPLA_2_-α production, resulting in reduced AA required for PGE generation. cPLA_2_-α dependence of PGE_2 _production in IL-1β-stimulated RA SF has previously been reported [34]. Studies in sPLA_2_-transfected HEK293 cells [51] and mesangial cells from cPLA_2_-*α*-deficient mice [52] suggest that sPLA_2 _can act along with cPLA_2_-α to maximize arachidonate release and increased PGE_2 _synthesis. A functional cross-talk between cPLA_2_-α and sPLA_2_-IIA in IL-induced RA SF cells, such as that observed in other cell types [51-53], may signify the importance of sPLA_2 _relative to cPLA_2 _induction in cytokine-stimulated RA SF cells and its inhibition by PIP-18 for RA treatment. Further work would be of benefit to determine whether these mechanisms occur.

The hTNF Tg197 model [16] used in this study is a clinically relevant model recommended by the US Food and Drug Administration for screening potential RA candidate drugs [54]. As compared with PIP-18, methotrexate and celecoxib are less potent; being able to suppress only synovitis, but not cartilage destruction and bone erosion to a significant extent. Because the efficacy of methotrexate is influenced by genetic factors, the reduced responsiveness of Tg197 mice to methotrexate may be related to adaptive immunity in arthritis development [21]. Ineffectiveness of methotrexate has previously been reported for Tg197 mice [21] and other arthritis animal models [22,55]. In contrast to the protective effect of celecoxib seen in various murine arthritis models [24,56], we did not find any reduction in the clinical scores of celecoxib-treated Tg197 mice, which express high levels of TNF mRNA and protein in their inflamed joints [16] and circulation [57]. Inhibition of COX-2 by celecoxib may exacerbate TNF production as a result of an imbalanced rise in thromboxane A_2 _relative to PGE_2 _levels [58], and the corresponding surge in TNF levels may provide an explanation for the reduced efficacy seen in Tg197 mice with celecoxib treatment.

AF-2, a 9-mer PLA_2 _inhibitory peptide derived from uteroglobin and annexin-1 amino acid sequences, shows potent anti-inflammatory activity in diverse animal models [59]. In Tg197 mice, it significantly (*P *< 0.05) moderates histopathologic score of synovitis, cartilage destruction and bone erosion (Figure 7), but fails to show appreciable abrogation of AS (Figure 5b). As observed previously in other studies [21,60], infliximab is also very effective in inhibiting inflammation and bone destruction in our study. No significant difference established between PIP-18 and infliximab for the total (Figure 6f) as well as differential histopathologic score on synovitis, cartilage, and bone (Figure 7) may seem to suggest equal efficacy between the two treatments. However, when the two drugs are compared in terms of molar basis, the efficacy of infliximab would nevertheless outweigh that of PIP-18. A statistically significant difference (*P *< 0.05, PIP-18 *vs *infliximab) noted between the two treatments on the AS (Figure 5b) is suggestive of the superior potency of infliximab relative to PIP-18 in reducing the disease activity.

It has been reported that TNF stimulates sPLA_2_-IIA gene expression and secretion by different transcriptional activation pathways [61]. High levels of TNF expressed in the inflamed joints of Tg197 mice [16] could facilitate sPLA_2 _expression and secretion, and amplify the available pool of sPLA_2 _that is highly expressed in the articular cartilage and chondrocytes of RA joints [62,63]. However, it should be noted that this speculation is based on the results obtained with murine mesangial cells [61], and may not be directly related to human SF cells. Besides stimulating sPLA_2_-IIA production, TNF is also capable of inducing cartilage catabolism via increased MMP expression and activation [64]. In Tg197 mice, PIP-18 significantly reduced serum levels of msPLA_2_, mIL-6, and hTNF-α as compared with untreated or vehicle-treated control animals. Considering that PIP-18 significantly reduces serum TNF-α levels in Tg197 mice, the possibility that MMP gene expression could also be an indirect effect of PIP-18 through suppression of TNF production should also be taken into account. From the data, it is plausible to suggest that PIP-18 suppresses p38 MAPK phosphorylation that in turn suppresses TNF production because cytokine production is regulated significantly by p38 MAPK, whereas MMP production is regulated both by p38 MAPK and JNK. It has been reported that blockade of TNF leads to a reduction of osteoclast numbers and enhanced osteoblast numbers [65]. Hence, the PIP-18 peptide may be a potential agent for preventing pathologic bone loss. Experimental studies to verify whether the peptide directly affects osteoclast precursor cells to suppress their differentiation to mature osteoclasts are currently underway. Although LY315920 and MMP-II inhibitors used in this study are well defined [25,26] and have been extensively used in several studies [29,30,66,67], the former is known for its varying potency for several isoforms of sPLA_2 _[28], while the latter is a broad-spectrum metalloproteinase inhibitor [26]. Hence, data obtained with such pharmacological agents should be interpreted with caution.

## Conclusions

In conclusion, our data show that PIP-18 significantly inhibits sPLA_2_-IIA enzymatic activity and downregulates sPLA_2_-IIA and MMPs (MMP-1, MMP-2, MMP-3, MMP-9) at both the transcript and the protein level in IL1-β-induced RA SF cells via attenuation of p38 MAPK phosphorylation. Treatment of TNF-driven Tg197 transgenic mice with PIP-18 significantly modulates disease progression by suppressing arthritis indicators (synovitis, cartilage and bone erosion) as well as circulatory levels of murine sPLA_2_, IL-6, and human TNF-α. The *in vitro *and *in vivo *preclinical data available from the present study thus validate the potential of this peptide as RA therapeutics.

## Abbreviations

AF-2: antiflammin-2; ANOVA: analysis of variance; AS: arthritis score; BSA: bovine serum albumin; cPLA_2_: cytosolic phospholipase A_2_; cpm: counts per minute; DMARD: disease-modifying anti-rheumatic drug; DMEM: Dulbecco's modified eagle medium; DMSO: dimethyl sulfoxide; ELISA: enzyme-linked immunosorbent assay; ERK: extracellular signal-regulated kinase; FBS: fetal bovine serum; GAPDH: glyceraldehyde 3-phosphate dehydrogenase; hr: human recombinant; IL: interleukin; JNK: Jun N-terminal Kinase; MAPK: mitogen-activated protein kinase; MMP: matrix metalloproteinase; MMP-II: matrix metalloproteinase inhibitor-II; NF: nuclear factor; OA: osteoarthritis; PBS: phosphate-buffered saline; PGE: prostaglandin; PIP: phospholipase inhibitor from python; PLA_2_: phospholipase A_2_; RT-PCR: real-time polymerase chain reaction; RA: rheumatoid arthritis; sPLA_2_-IIA: secretory phospholipase A_2_-group IIA; SF: synovial fibroblast; TIMP: tissue inhibitor of metalloproteinase; TNF: tumor necrosis factor.

## Competing interests

PG, M-MT, PVK and PA are all employees of the National University of Singapore, which supports the research project and finances this manuscript (including the article-processing charge). ED and GK are employees of the Institute of Immunology, Biomedical Sciences Research Center, Greece. PG and M-MT have applied for the patents relating to the content of this manuscript: Phospholipase A_2_-inhibitory peptide with anti-arthritic and neuroprotective activities (US Patent: 7,176,281); Methods and Compositions for Treatment of Arthritis and Cancer. US Patent Application: 20070037253 Filed: April 28, 2006 and is now under examination). PVK, PA, ED and GK declare that they have no further financial competing interests. All authors declare that they have no non-financial competing interests.

## Authors' contributions

M-MT carried out all aspects of the study, including the initial study design, experimental work, data analyses, graphics, and wrote the manuscript. ED was substantially involved in the coordination of the study, participated in animal experiments, and also in the layout and reviewing of the manuscript. PA performed the real-time PCR and cell-based assays, and participated in respective data analyses. GK established the Tg197 arthritis model and provided logistical support and intellectual contributions. PVK performed preclinical analyses and provided clinical specimens. PG contributed to conception and design of the project, and organized for collaborative research with ED and KG, discussed the data with the first author M-MT, and provided intellectual contributions.
